# Fried green burgers: a promising food for plant-based dieters. Ingredient characterization, processing, and future development

**DOI:** 10.1007/s13197-025-06463-0

**Published:** 2025-11-10

**Authors:** Amr E. Edris, Heba H. Salama

**Affiliations:** 1 Aroma and Flavor Chemistry Department, Food Industries and Nutrition Research Institute, National Research Centre, Cairo, Egypt; 2 Dairy Department, Food Industries and Nutrition Research Institute, National Research Centre, Cairo, Egypt

**Keywords:** Legume-based burger, Falafel, Ingredient, Nutritional-antinutritional factors, Deep fat frying, Development in the frying process

## Abstract

Currently, the world witnesses a growing interest toward conversion to a plant-based diet due to some behavioral or nutritional reasons. The fried green burger (FGB), which is globally known as “falafel” is an example of a flourishing plant-based burger in which meat is substituted with one of the legume seeds (which is not an oilseed) and processed by deep-fat frying. This plant-based food originated in the Middle East and some Asian regions many years ago, then spread to the Western world in the 20th century due to increased immigration. Made entirely from plants, FGB’s appealing taste and relatively low price make it a potentially attractive meal option for dieters. In this review, the authors will discuss fundamental elements pertaining to the FGB. This includes the various types of plant ingredients employed in processing, along with their nutritional, anti-nutritional, and sensorial characteristics. The review examines various processing steps, including deep-fat frying. It discusses the challenges of the frying process, such as oil uptake and the generation of hazardous compounds that develop during this process. Additionally, it reviews developments aimed at improving the quality of (FGB) during frying.

## Research highlights

Plant-based fried green burger.

Legumes as a main element for making fried green burger.

Nutritional and anti-nutritional characteristics of legumes.

The frying process and its effect on flavor, nutritional value and health.

Future development in the frying process to mitigate drawbacks.

## Introduction

The idea of a plant-based diet dates back to the ancient ages, either as part of established community traditions or as a part of personal doctrines or preferences. However, the concept of a plant-based diet varies widely in its definition. It can mean eating a diet rich in a large proportion of plant-dominant foods (Storz 2022). Another definition indicates that a plant-based diet should exclude all animal products and flesh foods like meat, birds, and fish, as in the case of veganism. Some other diets exclude meat or poultry but include fish, as in the case with the pesco-vegetarian diet. On the other hand, an ovo-lacto vegetarian diet excludes meat, fish, or poultry but includes eggs and milk (Gehring et al. 2021). Therefore, the inconsistent usage of the term “plant-based diet” may cause significant confusion and make comparisons of studies difficult.

Consumers who change their eating behavior and convert from an omni-diet (omnivores) to a plant-based diet have different motives for such conversions (Kopplin and Rausch 2022). Among these are health concerns, social concerns, and animals’ welfare. While others also have environmental concerns, they consume low environmental-impact food that does not increase greenhouse gases. There are many comprehensive articles that cover different aspects of plant-based diets and how they will affect the future of food products and markets (Tachie et al. 2023; Storz 2022; McClements and Grossmann 2021).

Plant-based meat analogues, are extensively investigated and commercialized in the food market (Ishaq et al. 2022). Burger patties are some of the most common plant-based meat analogues. They are typically made from a combination of protein-rich plant sources, such as peas and soybeans (Marchi et al. 2021), as well as a mix of mushroom-soy protein isolate and chickpea flour (Mazumder et al. 2023).

**Fig. 1:**
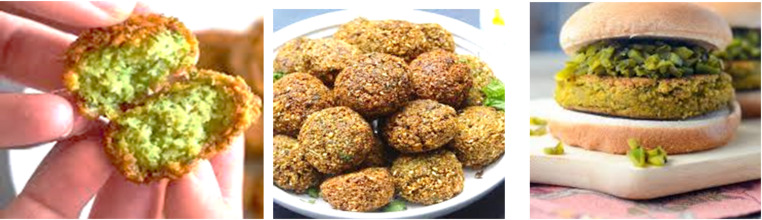
Fried green burgers

These kinds of plant-based burgers are cooked by grilling, just like ordinary meat-based burgers. On the other hand, there is another type of plant-based burger patty made entirely from legumes and processed by deep-fat frying, which is called a fried green burger (FGB). This nomenclature is not coined or proposed by a scientific food committee or meetings, but it is trending among the general public due to its interior vegetative green color and burger-like shape (Fig. 1). The name FGB is also used synonymously with “falafel”, which is famous among the people in the fast-food street markets around the world.

The authors of the current review observed a lack of scientific and conclusive information about FGB in the literature. Based on that, detailed elements related to the plant ingredients of FGB, their nutritive value and anti-nutritive and sensory characteristics will be tackled. The review will also exhibit the frying process of FGB and its role in flavor development. The discussion focuses on the formation of health risk components related to frying, as well as recent opinions aimed at reducing the creation of these compounds.

## Characterization of plant ingredients used for making FGB

### Legume’s ingredients, their nutritional facts, and health benefits

**Fig. 2:**
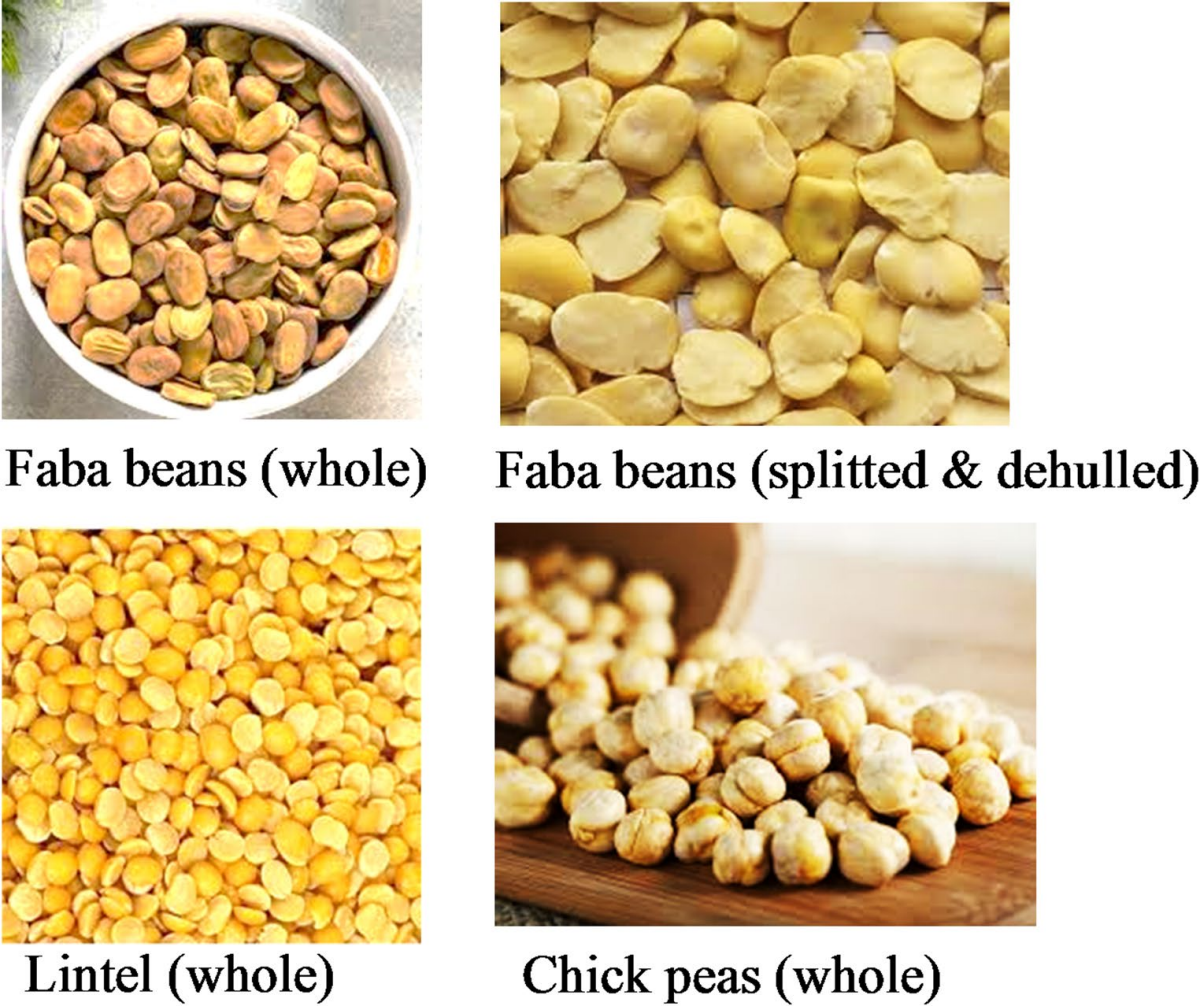
Main legumes used for making FGB

FGB is composed principally of a legume ingredient (which is not an oilseed), such as dehulled faba beans (*Vicia faba*) cotyledon, chickpeas (*Cicer arietinum*), which are also called kabuli chickpeas or garbanzo beans, and lentils (*Vicia lens)*, (Fig. 2).

Legumes belong to a plant family called Fabaceae (or Leguminosae). They are considered a rich source of plant-based proteins due to their ability to fix atmospheric nitrogen into their tissues. That takes place through bacteria nodules in their roots, such as Rhizobia (Bustos-Segura et al. 2024). The percentage of proteins in the three main legumes that are used for making FGB is 22.7–34.7% for faba beans (Martineau-Côté et al. 2022), 21% for chickpea, and 10.5–27.1% for lentils (Singh et al. 2022), on a dry weight basis. Protein percentages of the main legumes in FGB is important for understanding the nutritional composition of this product in comparison to meat-based burgers.

Different essential amino acids are present in legumes, with the highest average values of arginine, glutamic acid, and threonine found in faba beans. On the other hand, chickpeas stood the highest in threonine and methionine (Mecha et al. 2023). It is worth indicating that faba beans and chickpeas are the common legumes used for making FGB in the Middle Eastern region, while lentil-made FGB is more likely to be common in India and Pakistan.

On the other hand, traditional animal-based burgers are characterized by higher protein contents compared to legume-based burgers (Higuera et al. 2021). Besides, there are some other differences in nutritional composition (Swing et al. 2021) and protein quality and digestibility (Cutroneo et al. 2023) among the two burger types. One should take into consideration that legumes are an incomplete source of protein due to the lack of sulfur-containing amino acids.

Besides proteins, legumes are also rich sources of vitamin B, fibers, and minerals like zinc, potassium, and magnesium (Hughes et al. 2022). However, meat-based burgers are higher in mineral content, especially iron, potassium, phosphorus and zinc (Higuera et al. 2021). The significant value that characterizes legume-based burgers is their cholesterol-free nature, low saturated fat content, and low glycemic index (Di Cairano et al. 2020). More details about the functional properties, nutritional value, and health benefits of legumes were discussed in another investigation (Serrano-Sandoval et al. 2023).

Unfortunately, legumes contain some anti-nutritional factors that reduce their digestibility and, consequently, their nutritional values. These factors include tannins, phytic acids, and others. Tannins, for example, bind to proteins and affect the bioavailability of amino acids, in addition to inhibiting the activities of digestive enzymes (Singh et al. 2023). The amount of tannins varies depending on the type of legume, with faba beans being the highest in tannin content (Sorour et al. 2018), followed by chickpeas (Idate et al. 2021), then lentils (Galgano et al. 2021). However, there is a novel cultivar of faba beans that is characterized by containing zero tannin (Nyende et al. 2023).

Besides tannins, legumes also contain phytic acid, which acts as an anti-nutrient factor. It can impair the body’s absorption of some minerals like iron, calcium, and zinc, leading to mineral deficiency over time (Wang and Guo 2021). That is besides inhibiting the biological functions of food proteins and digestive enzymes like trypsin, causing digestive problems due to that anti-proteolytic action (Wang and Guo 2021). In addition to tannins and phytic acids, there are also some other anti-nutritional factors present in legumes related to FGB making. That includes oligosaccharides, lipoxygenases, hemagglutinins, and anti-proteases, which are reviewed in detail elsewhere (Ozolina et al. 2023).

Fortunately, anti-nutrient agents in legumes are water soluble. Thus, their concentration can be significantly reduced by soaking the seeds in water for 12–16 h (Sorour et al. 2018; Abd El-Moniem et al. 2000; Samtiya et al. 2020). During soaking time, water activates some endogenous enzymes like phytase, which consequently reduce the amount of phytate in legumes.

It is noteworthy that prior to the identification of anti-nutrient components in legumes, soaking in water was commonly the first processing step for making FGB. That is intended to soften the legume seeds and facilitate the grinding process into a smooth legume paste (or dough) with a moisture content of about 50%-55% prior to molding and frying (Ziena 2007).

### Aromatic plant ingredients in FGB and their sensory attribute

Aromatic plants, such as herbs and spices, serve as minor but essential ingredients in the processing of FGB due to their contribution to the sensory characteristics of the product. That is because aromatic plants contain a volatile oil fraction, which varies between 0.1% and 4.0%. These oils comprise several volatile terpenes and sulfur-containing compounds, which, along with the frying process, contribute to the overall flavor experience of FGB. Aromatic plants are mixed with the water-soaked legume seeds and ground together into a soft green paste. This paste captures the aroma of the volatile compounds that are released from the aromatic plants during the grinding process. Table (1) shows examples of the aromatic plants ingredient that are commonly used to contribute to the flavor of FGB. The volatile oils of leeks, onions, and garlic are rich in some major sulfur-bearing volatile compounds (Table 1).,. These compounds possess a distinctive sharp, pungent, and sulphurous aroma (Jameson 2009) which characterize the original flavor of FGB.

**Table 1:** Aromatic plants used in making fried green burger and their major volatile compounds

Aromatic plant	Latin name	Major volatile compound*	References
Leeks	*Allium borrum*	dipropyl disulfide; dipropyl trisulfide; and diallyl disulfide	Gargi et al. (2024)
Onions	*Allium sepa*		
Garlic	*Allium sativum*		
Parsley	*Petroselinum crispum*	apiol	Franciscato et al. (2022)
Dill	*Anethum graveolens*	α-phellandrene & limonene	Cătunescu et al. (2023)
Coriander	*Coriandrum sativum*	linalool	Al-Khayri et al. (2023)
Sweet fennel	*Foeniculum vulgare*	*trans*-anethol	Šunic et al. (2023)

*These components are found as major constituents in the volatile oil (essential oil) of each corresponding aromatic plant

On the other hand, fresh herbs like parsley and dill give FGB paste its characteristic green color, from which “green burger” is coined, due to their chlorophyll content. Although they do not contain volatile sulfur compounds, they possess a strong terpenic taste due to some volatile compounds like apiol in parsley and α-phellandrene and limonene in dill (Franciscato et al. 2022; Cătunescu et al. 2023).

Besides the above-mentioned fresh herbs, seeds of dry spices like coriander and sweet fennel are also added to the ground legume paste just before frying. The volatile oil of coriander contains linalool (60%-80%) as a major constituent (Al-Khayri et al. 2023). This compound is characterized by a spicy, woody aroma, which contributes to the flavor of FGB. On the other hand, fennel seeds contribute a sweet, anise-like aroma with herbaceous undertone. That is due to *trans*-anethole which can reach up to 75.5% of the composition of sweet fennel volatile oil (Šunic et al. 2023). On the other hand, it is important to note that certain fennel seed species contain a volatile oil fraction that is high in another compound known as estragole (up to 60% of the oil’s composition) (Abdellaouia et al. 2020). Fennel seeds belonging to this species are not recommended for application in FGB due to the serious health hazard of estragole Bergau et al. 2021).

### Processing of FGB

**Fig. 3:**
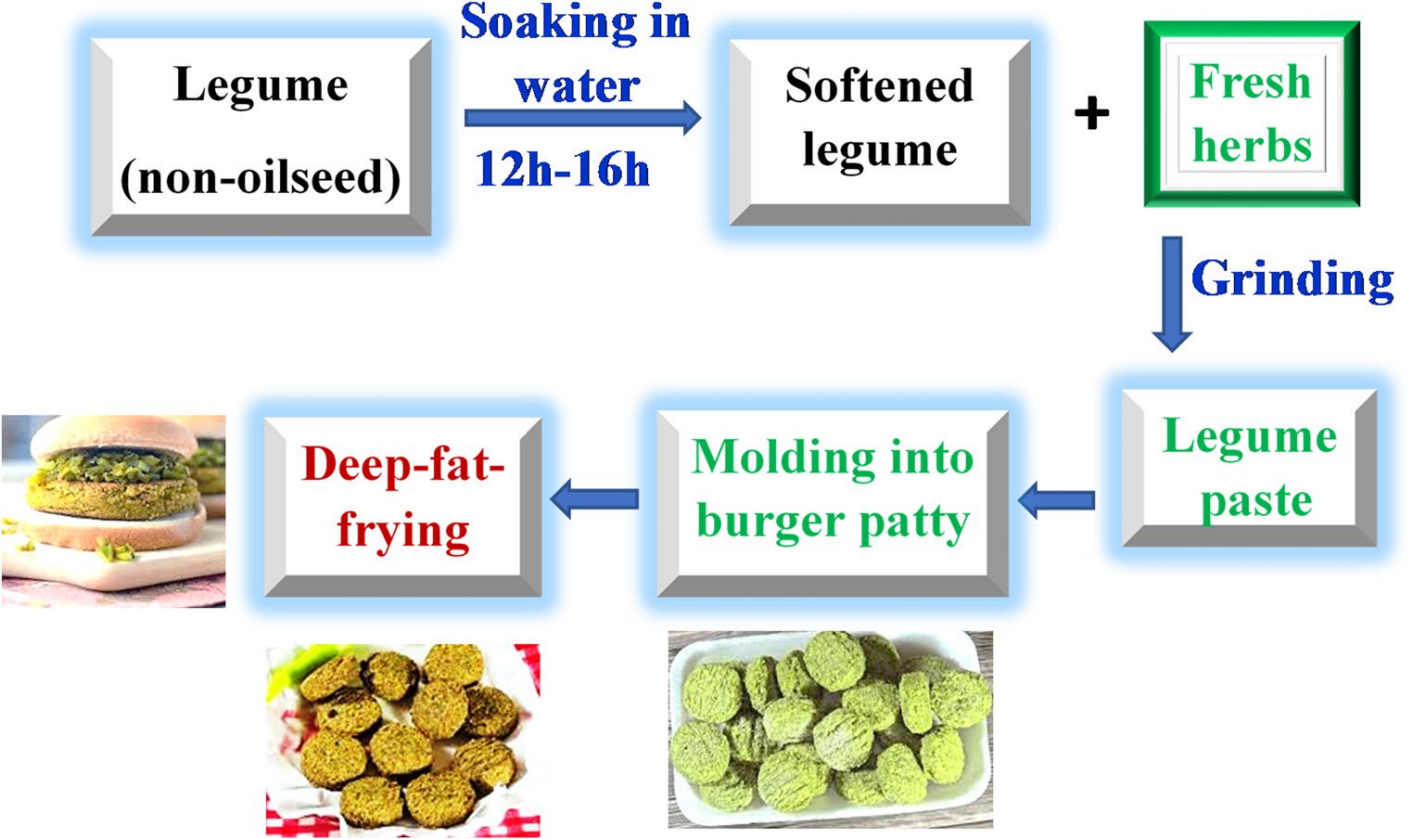
Schematic representation of FGB processing

Figure 3 summarizes the various processing steps involved in the manufacture of FGB. The following passages will discuss each of these steps in detail.

### Preparation of legumes for the frying process

The processing of FGB starts with soaking the legume seeds in water for 12–16 h to induce softening (Sorour et al. 2018; Abd El-Moniem et al. 2000). Water absorption during that treatment was found to be 148.6% in the case of faba beans (Abd El-Moniem et al. 2000). After that, the softened seeds are mixed with the fresh herbs and ground into a soft, fluffy green paste. The moisture content of the paste is usually about 50–55% (Ziena 2007). Once the legume paste is prepared, it becomes ready for the burger patty molding and the final frying step.

It is noteworthy that, due to the high moisture content of the ground legume paste, its shelf life is short, and it is susceptible to microbial contamination unless fried in a short period of time. One of the classical approaches to increasing the shelf life of the paste is to dehydrate the freshly prepared legume paste into instant powder (Ziena 2007). The powder can then be rehydrated with water just before frying, without concern for microbial contamination. Most recently, phenolic volatile extracts from aromatic plants like eugenol and carvacrol were added to the fresh ground legume paste as natural antimicrobial preservatives (Olaimat et al. 2024).

### Deep-fat frying process

Unlike meat-based burgers or plant-based meat analogue burgers, which are processed by grilling, legume-based green burgers (FGB) are processed by deep-fat frying in vegetable oil. This treatment leads to the development of unique sensorial attributes that characterize FGB, like flavor and crispiness, that distinguish FGB from other plant-based burgers. All types of vegetable oils can be used in the frying process depending on their availability and economic feasibility. The frying of the molded ground-legume burger patty takes place at 175–180 °C for about 3–5 min (Angor 2023).

During the deep-frying process, different thermal reactions and interactions take place between the legume and the hot oil, leading to changes in the overall flavor, texture, nutritional, and functional properties of any fried food material (Sivaranjani et al. 2024). Regarding flavors, the distinctive taste of FGB originates from the interaction of its legume components, along with herbs and spices, with the frying oil. The high temperature of frying initiates a number of thermally induced reactions, like the browning reaction “Maillard reaction,” lipid oxidation, hydrolysis, and amino acid degradation, in addition to caramelization (Sivaranjani et al. 2024). As a result, a variety of aliphatic aldehydes, ketones, alcohols, and heterocyclic aroma and flavor compounds are generated, which typically characterize the flavor of deep-fat fried food, including FGB (Wang et al. 2023). What is missing in this context is the need for a study that is directed toward the isolation and identification of the key volatile aroma compounds generated in FGB during frying. One should take into consideration that frying oil is considered a perfect medium for carrying and enhancing the fatty and nutty flavors of fried foods in general.

Beside flavor generation, the deep-fat frying process is also accompanied by a change in the texture attributes of food (Wang et al. 2023). For instance, in the case of FGB, the soft, fluffy paste of legume seeds transforms during frying to a product that is crispy. That is due to the alteration of the physical properties of the raw legume material during frying as a result of well-known chemical reactions, including legume protein denaturation (Xie et al. 2022) and legume starch gelatinization, among other reactions (Wang et al. 2023).

Regarding the effect of deep-fat frying on the nutritional value of FGB, we would like to indicate that heat processing in general has been reported in earlier studies to increase protein digestibility of legumes (Jood et al. 1989). That takes place by destroying heat-labile protease inhibitors and also by denaturing globulin proteins.

Focusing on deep-fat frying of legumes, particularly broad beans (faba beans), frying led to considerable loss of total oligosaccharides accompanied by loss of about 17% lysine and only 7.5% of total amino acids (Abd El-Moniem et al. 2000). In addition, the frying process led to a greater ratio of Leu/Lys in comparison to the starting raw material.

Heat treatment of legumes, like frying, also led to a decrease in the content of anti-nutritional factors such as phytate (Ozolina et al. 2023; Samtiya et al. 2020). For instance, faba beans, which are the major legume utilized for making FGB, had a total loss of phytate content of 67% after frying (Abd El-Moniem et al. 2000). Consequently, that led to an improvement in protein digestibility. It is worth noting that soaking legumes in water as a pretreatment before frying also plays a major role in reducing phytates, as we described earlier in this review (Sorour et al. 2018).

## Health challenges and side effects accompany the deep-frying process of FGB

### Generation of potential carcinogenic compounds

In the previous passages, we reviewed the role of the frying process in the generation of flavor and crispy texture in FGB. The drawbacks of frying will also be covered in this part because it produces several hazardous compounds that might cause health problems, such as acrylamides and heterocyclic aromatic amines (HAAs).

**Fig. 4:**
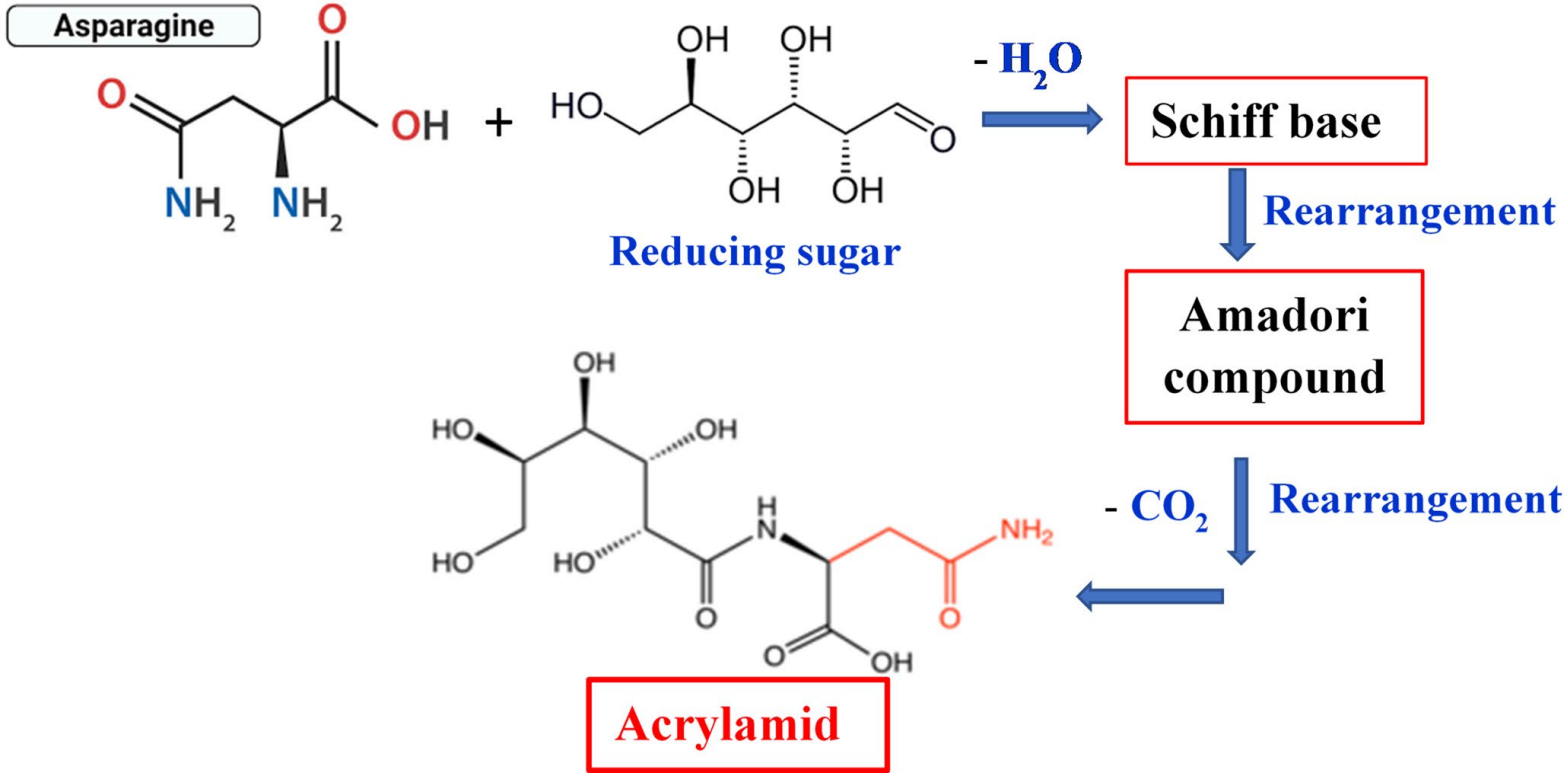
Formation of acrylamide in FGB

Acrylamide is a carcinogenic compound developed in heat-treated food via the Millard reaction (Adimas et al. 2024). Deep-fat frying of starch-rich food materials is a typical environment for the formation of acrylamide (Soliman and Hamed 2022). This compound is thought to be formed during the frying process through the reaction between asparagine from the protein component of food with the carbonyl compounds from the carbohydrate component, (the reducing sugar, Fig. 4). A comprehensive study describing the potential cancer risk associated with dietary acrylamide exposure is available (Basaran et al. 2023).

Regarding acrylamide in FGB, an early investigation (Al-Dmoor et al. 2004) found that the average amount of this compound in green burgers fried at 170 °C depends on frying time. During 4 min of frying, the amount of acrylamide was about 1000 µg kg^− 1^. On the other hand, increasing frying time to 8 min increased acrylamide content radically to ~ 4200 µg kg^− 1^ (Al-Dmoor et al. 2004). A most recent study (Abdalla et al. 2023) indicated that the content of acrylamide from samples of FGB collected from the market of Jordan was 106 ± 21 µg kg^− 1^. So, it is clear that the temperature and time of frying control the amount of acrylamides in FGB.

Heterocyclic aromatic amines (HAAs) are another class of carcinogenic compounds that are also generated from Maillard reaction pathways during the frying of protein-rich food, especially meat (Zhao et al. 2023). The presence of (HAAs) in FGB has not yet been studied, as indicated by our literature search on the topic. However, due to the high protein content of legumes (24.5–35%), it is possible that HAAs may form in FGB.

### Oil uptake (absorption) during the frying process

Oil absorption during food frying is a common phenomenon and represents a major concern to the fried food industry due to the high fat content of the fried food (Zhang et al. 2024). Evaporation of water in the form of vapor due to the high temperature of the frying process leaves voids in the frying material, which let the oil migrate into it. The mechanism of this process was discussed in details by different investigators (Devi et al. 2021; Zhang et al. 2020). Oil uptake in fried foods, including FGB, raises some health concerns that are linked to obesity and cardiovascular diseases due to the high caloric content of oils (Li et al. 2025; Duan et al. 2025; Djouss et al. 2015). Additionally, it is important to consider the economic and capital losses resulting from excessive and unnecessary oil consumption. An early investigation (Abdullah 2015) delt with reducing the oil uptake in FGB by spraying the surface of the molded legume patty or immersing the legume paste as a whole before frying in a solution of hydrocolloid like gum Arabic, CMC, or pectin. The oil reduction according to this treatment reached 14% to 25% for spraying versus immersing the ground legume paste in the hydrocolloid solution, respectively. More research that can lead to developments in the processing of FGB by reducing acrylamide formation and oil uptake during frying is going to be reviewed in the coming section.

### Future development in the processing of FGB

The main endeavors toward the development of FGB lie in improving the deep-fat frying process. Like in many other fried foods, researchers tend to improve the frying process by minimizing the formation of acrylamides and by reducing oil uptake (fat content).

Generally speaking about progress in acrylamide control during frying, Zhu et al. (2024) used pectin and hydrolyzed pectin coating as pre-frying treatments to reduce acrylamide formation in potato chips. Another approach includes soaking food materials (potato slices) in tannic acid solution prior to frying, which can significantly reduce the amount of acrylamide (Soliman and Hamed 2022).

Concerning FGB, Mahfouz et al. (2019) found that mixing turnip root powder at 5.0 wt% with ground legume paste before frying can reduce acrylamide formation in FGB at 170 °C for 4 min. That happened in parallel with increasing other healthy biological parameters of FGB, like flavonoids, total phenols, glutathione, and antioxidant properties. Acrylamide in FGB can also be reduced by dipping the legume paste into a 1% pectin coating solution with or without the transglutaminase enzyme, which was added to the legume paste before frying (Al-Asmar et al. 2019). Results showed that pectin coating alone was effective in reducing the acrylamide level significantly, up to 59.3%, which can be further reduced to 84% by using both pectin solution and transglutaminase mixed with the paste at 20 U/g. Transglutaminase can crosslink proteins in FGB, leading to improvements in the physiochemical properties and also decreasing oil uptake during frying.

On the other hand, controlling the oil uptake during the frying of green burgers was investigated using edible coating films made of dried fruit peels (Angor 2023). Results showed that dipping the ground legume paste patty in a coating film solution containing 5% of the fruit peel (orange peel albedo or apple peel powders) before frying was effective in reducing the oil uptake. However, orange peel albedo powder showed a greater reduction in oil uptake compared to apple peel powder. The mechanism of action of hydrocolloid or any other coating material is by forming a barrier that prevents moisture loss in the form of water vapor during frying. As a result, there will be no voids in the fried material that allow the migration of oil.

Parallel to this trend, other alternative frying media, like oleogel as a sole frying medium, were also investigated for reducing oil uptake during deep-fat frying (Mahmud et al. 2023). Some pretreatments that can modify the microstructure and reduce oil uptake in fried materials were also explored. That includes hot air drying combined with ultrasound treatment (Dehghannya and Ngadi 2023), along with other pretreatments like blanching, freezing, pulsed electric fields, microwaving, etc. (Zhang et al. 2024). However, these investigations were not specifically designed for FGB per se; they were generalized to different fried foods like potato chips.

In addition to the previous approaches, the air-frying technique was also developed to reduce oil uptake during the frying process. This technique allows the circulation of superheated air around the food product with little or no oil instead of full immersion in hot fat (Tellez-Morales et al. 2024). That technique was also applied for frying green burgers (Fikry et al. 2021, 2022). The results indicated that the oil content of the air-fried FGB was reduced by 45% at optimal frying conditions compared with the traditional deep-fat-fried control samples. Moreover, the air-fried FGBs were much crispier than the traditional fried control sample.

## Conclusion

The fried green burger (FGB) is a promising plant-based food product designed to meet the needs of a significant segment of consumers, particularly vegetarians, vegans, and individuals transitioning to a plant-based diet.

The information presented in this overview illustrated basic facts and details about FGB, which could be a platform for further contributions. More research work is anticipated in the future to overcome some processing hurdles, especially in the frying process, for securing a safer and healthier plant-based food product.

## Data Availability

Not applicable.
